# Superselective renal artery embolization for treatment of urological hemorrhage after partial nephrectomy in a solitary kidney

**DOI:** 10.1590/1677-5449.200005

**Published:** 2020-10-23

**Authors:** José Maciel Caldas dos Reis, Fábio Akimaro Kudo, Moisés do Carmo Bastos, Humberto Balbi Reale, Maurício Figueiredo Massulo Aguiar, José Victor Figueiredo dos Santos

**Affiliations:** 1 Hospital Amazônia, Serviço de Cirurgia Vascular e Endovascular, Belém, PA, Brasil.; 2 Hospital Amazônia, Serviço de Urologia, Belém, PA, Brasil.; 3 Centro Universitário Metropolitano da Amazônia – UNIFAMAZ, Belém, PA, Brasil.

**Keywords:** kidney, renal hemorrhage, kidney angiography, therapeutic embolization, rim, hemorragia renal, angiografia renal, embolização terapêutica

## Abstract

Embolization is a well-known and accepted form of treatment for bleeding caused by a multitude of renal procedures. We present a case of a 66-year-old woman who had a history of left nephrectomy for clear cell carcinoma seven years previously and now presented with a 6 cm tumor involving the solitary kidney. She underwent partial laparoscopic nephrectomy with removal of the tumor on the right kidney. In the immediate postoperative period she had important and persistent hematuria associated with tachycardia, hypotension, and lumbar pain. After showing signs of hemodynamic instability, she was taken to the catheter laboratory where selective angiography of the right kidney was performed. Superselective embolization with controlled release of fibrous microcoils was performed. The superselective renal embolization technique performed on an emergency basis to control hemorrhage after a urological procedure is effective and achieves lasting resolution of symptoms.

## Resumo

A embolização é uma forma bem conhecida e aceita de tratamento para sangramento de inúmeros procedimentos renais. Apresentamos o caso de uma mulher de 66 anos de idade com história de nefrectomia esquerda por carcinoma de células claras havia 7 anos, que desta vez apresentava rim único afetado por um tumor de 6 cm. Ela foi submetida a nefrectomia parcial videolaparoscópica com remoção do tumor no rim direito. Apresentou, no pós-operatório imediato, hematúria importante e persistente associada a taquicardia, hipotensão e dor lombar. A paciente apresentou sinais de instabilidade hemodinâmica e foi levada ao setor de hemodinâmica, onde foi realizada angiografia seletiva do rim direito. Foi localizado foco sangrante, sendo então realizada embolização superseletiva com liberação controlada de microcomolas. A técnica de embolização renal superseletiva realizada em caráter de emergência para controlar a hemorragia após um procedimento urológico é uma solução eficaz, minimamente invasiva e segura para controle dos sintomas.

## INTRODUCTION

Renal hemorrhage is a life-threatening situation that can be caused by several urological procedures.^1^^,^^2^ Conservative treatment, managed by endovascular therapy, is regarded as the preferred approach for most cases, because surgical exploration may result in nephrectomy.^1^

First described by Lalli and Peterson in 1969, renal transarterial angioembolization is now a well-established endovascular technique. Development of innovative materials and improved surgical techniques have allowed it to be employed in a preventive manner in vascular defects, cancer, and even for therapeutic embolization of iatrogenic and spontaneous hematuria or after urological procedures.^2^^,^^3^

The main criteria for choosing the technique are minimal invasiveness, low morbidity and mortality rates, few early or late complications, lack of need for general or intrathecal anesthesia, and low interference in renal function.^3^^-^5

The objective of this article was to report the effectiveness of superselective angioembolization of the renal artery in a case of hemorrhage in the urinary tract after partial nephrectomy in a patient with a solitary kidney.

## PART I – CLINICAL SITUATION

**Figure 1:**
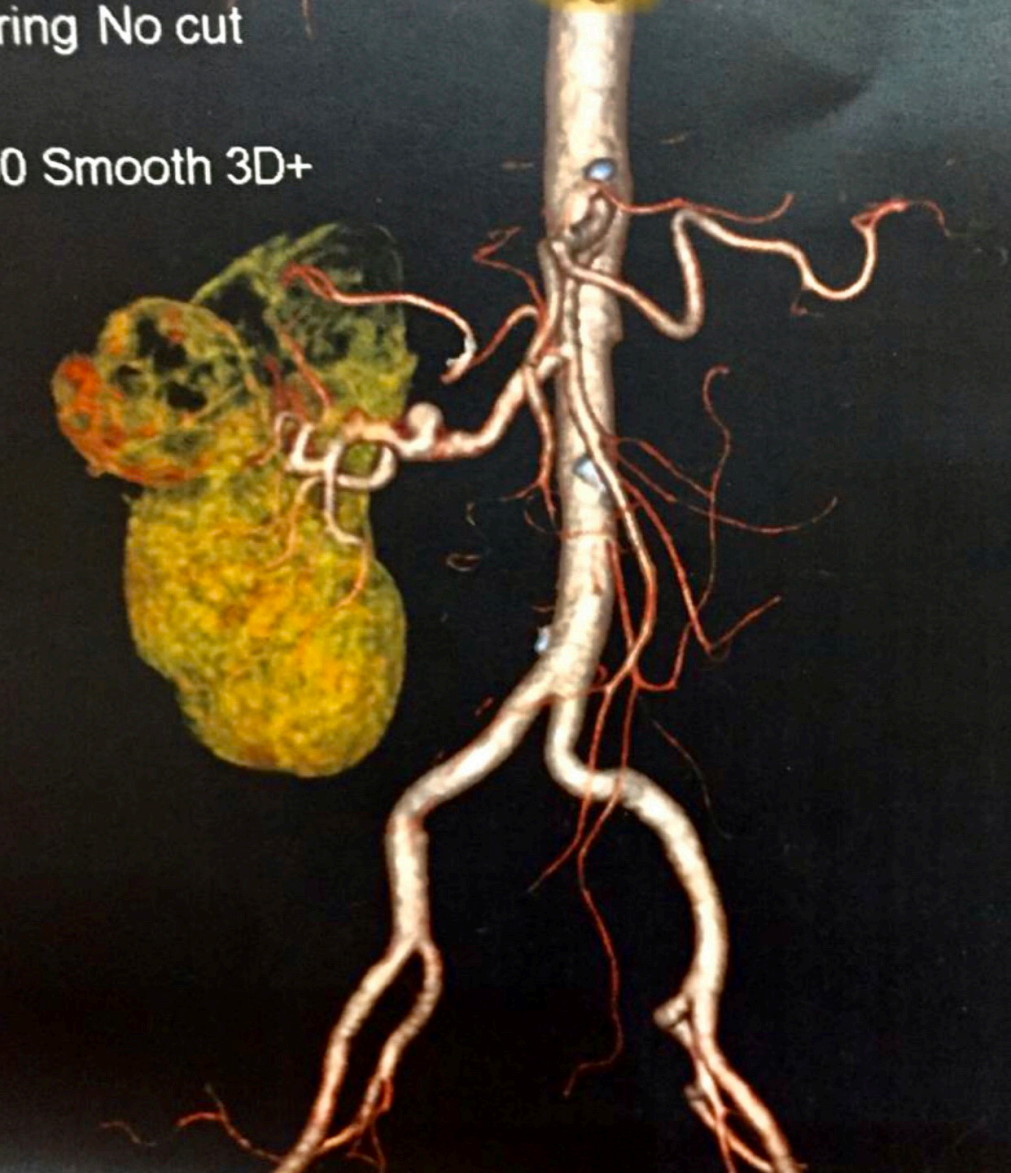
Preoperative computed tomography reconstruction showing tumorous lesion and renal artery aneurysm.

**Figure 2:**
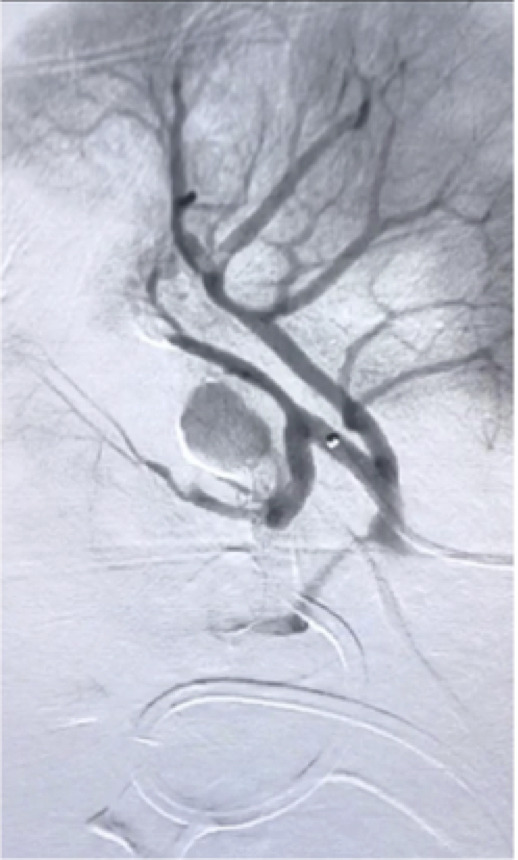
Superselective catheterization of the interlobular artery showing pseudoaneurysm in a 66-year-old woman after videolaparoscopic partial nephrectomy.

We report the case of a 66-year-old woman who was hypertensive, dyslipidemic, and an ex-smoker who had undergone left nephrectomy for clear cell carcinoma seven years previously, and then presented on with a 6 cm tumor involving the solitary kidney affected and a 1.4 cm aneurysm of the middle third of the renal artery (Figure 1). She underwent partial laparoscopic nephrectomy with removal of the tumor from the right kidney (Figure 2). In the immediate postoperative period she developed important and persistent hematuria associated with tachycardia and lumbar pain.

In view of this presentation, the following treatment options were immediately considered: conventional surgical treatment with residual nephrectomy or endovascular treatment with embolization of the segmental renal artery with detachable coils.

## PART II – WHAT WAS DONE

**Figure 3:**
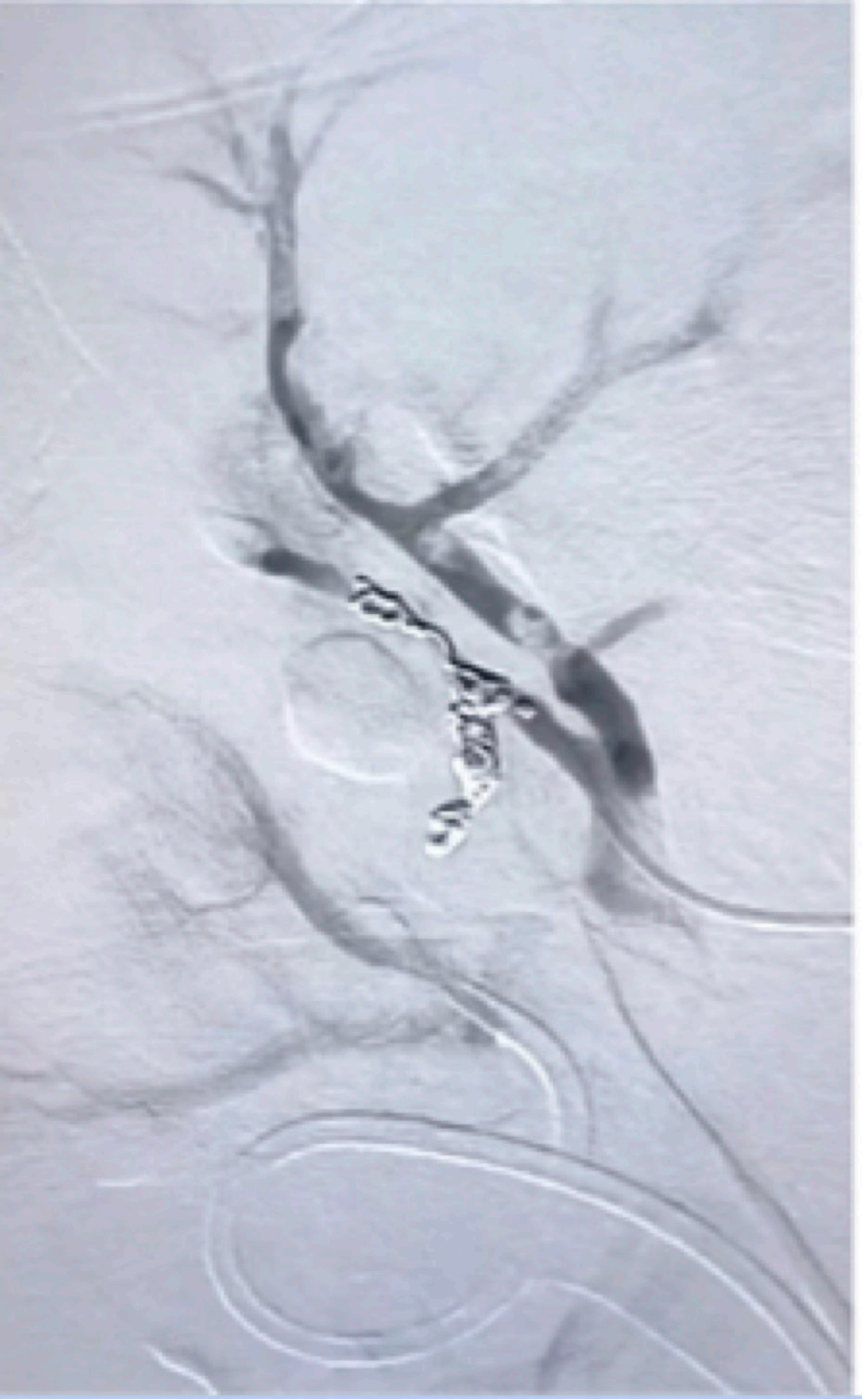
Microcatheterization of the offending vessel after post-superselective embolization angiography showing complete occlusion with metallic coils.

**Figure 4:**
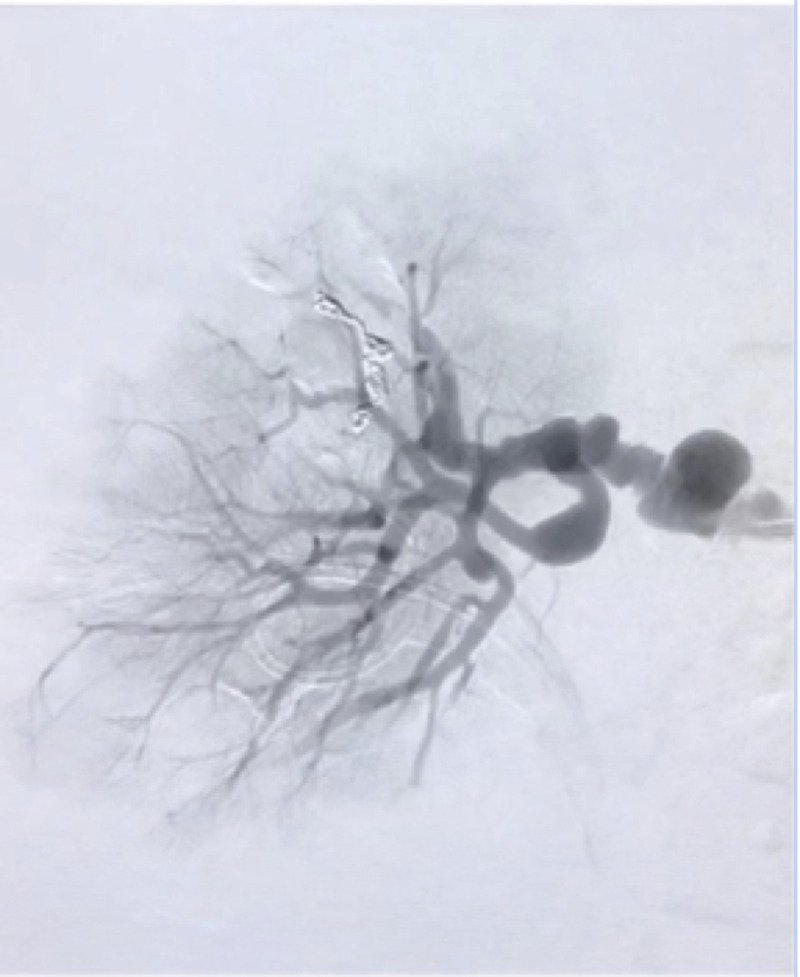
Selective arteriography of the right renal artery after effective embolization showing complete occlusion with metallic coils.

The patient was taken to the hemodynamics department and underwent intervention. Briefly, the right common femoral artery was punctured with the aid of ultrasound and a 5-F introducer sheath was inserted. Right segmental renal artery angiography identified active bleeding at a site suggestive of a pseudoaneurysm and a 2.4-F superselective coaxial catheter was advanced as close as possible to the hemorrhagic branch for selective and superselective renal angiography (Figure 3). Repeated angiography was performed to ensure the catheter was correctly positioned. Embolic material composed of controlled release fibrous microcoils (*Micromola fibrada Concerto – EV3*) was released into the affected branch. The degree of occlusion was determined with angiography after each microcoil deployment (three units). After total occlusion of the bleeding, a post-embolization renal angiography was performed to reveal a small avascular segment, preserving all vascularization to the remaining kidney (Figure 4). The procedure was uneventful, the hematuria ceased immediately after the intervention, the patient’s blood pressure levels stabilized, and there was no need for additional blood transfusions. She remained in the intensive care unit for observation and was discharged from the unit after 48 hours. On the first postoperative day (POD), elevation of nitrogenous wastes was observed without the need for renal replacement therapy and normalization occurred on the third POD. There were no complications during postoperative recovery in hospital. The patient was discharged from the hospital on the fourth POD and has been followed-up on an outpatient basis for four months, with no complications, and the renal artery aneurysm is being monitored.

## DISCUSSION

Treatment of renal vascular lesions depends on the etiology and clinical course of the disease. Hemorrhagic complications after urological procedures have been preferably treated by endovascular access since the 1970s, with applications in several clinical scenarios.^1^^,^^2^^,^^4^

Laparoscopic urological interventions have made it possible to perform numerous minimally-invasive diagnostic and therapeutic renal procedures, enabling “nephron-sparing” management. Nevertheless, the risk of renal vascular lesions with active bleeding, pseudoaneurysm, arteriovenous fistula, or a combination of these has remained unpreventable.^6^ In this case, the possibility of initially treating the renal artery aneurysm was discussed during the preoperative period, but the patient chose to resolve the tumorous lesion first.

Endovascular control by angioembolization is a minimally invasive and safe method with proven efficacy for management of hemorrhagic urological emergencies.^1^^-^5 Its potential for application even in critical patients makes it a first choice for attempted renal preservation, particularly in patients with a solitary kidney.^1^ Complications may occur occasionally following an embolization procedure, with a risk of inadvertent parenchymal infarction.^5^^-^7

Progressive advances in interventional radiographic techniques, such as enhanced imaging and the introduction of smaller devices, as well as more accurate embolic agents, have made superselective embolization of the renal artery an effective approach to diagnosis and treatment of renal hemorrhage.^1^^,^^2^ In general, it requires a short hospital stay, yields a rapid recovery, is usually performed without the need for general anesthesia and with low rates of early and late complications,^4^^-^6 limiting the classical surgical approach with nephrectomy to exceptional cases only.^3^^,^^4^

Various embolic agents have been described in the literature for controlling bleeding in the renal region, but most series report embolization with coils as the preferred technique for renal artery embolization in several clinical scenarios.^6^^-^10 This was the option chosen in the case reported here because it has some advantages compared to other methods. It is easy to manipulate, with controlled detachment into the target vessel only, promoting a minimal area of renal ischemia, since it does not close the distal microcirculation. Its dilution in the iodinated contrast and use of zoom capabilities during the injection allows accurate tracking of the embolic agent, but should be used with caution; once released, it cannot be repositioned and prevents access to more distal segments of the vessel into which it was implanted.^2^^,^^6^^,^^7^^,^^9^

Reported technical success rates vary between 71 and 100%.^4^^-^6 The result is clearly dependent on the degree of difficulty of the procedure in selected cases.^5^ Accordingly, in 2015, Thorlund et al.^5^ reported an overall technical success rate of 83% in a diverse group composed of 35 patients in whom superselective renal embolization proved to be a safe procedure associated with low morbidity and mortality, both when performed electively and in emergency situations.^5^

Sam et al.,^9^ obtained a technical success rate of 98% in 34 procedures, and a clinical success rate ranging from 83% to 98% at 24 and 72 hours respectively, without major or minor complications related to the procedure. In 2017, Ahn et al. conducted a systematic review including 79 publications between 2000 and 2016, encompassing the most diverse causes of renal bleeding and recommending conservative management with a minimally invasive approach as preferable to surgery, although suspicion of malignancy and hypovolemic shock suggest a conventional surgical approach as first option.^8^

In summary, the superselective renal embolization technique performed on an emergency basis to control hemorrhage after a urological procedure is effective, with immediate and lasting resolution of symptoms, aiding in maximum renal preservation particularly in patients with a solitary kidney.

## References

[1] Wang C, Mao O, Tan F, Shen B (2014). Superselective renal artery embolization in the treatment of renal hemorrhage. Ir J Med Sci.

[2] Góes AMO, Jeha SAH, Salgado JRC (2016). Embolização arterial superseletiva para tratamento de angiomiolipoma em paciente com rim único. J Vasc Bras.

[3] Ząbkowski T, Piasecki P, Zieliński H, Wieczorek A, Brzozowski K, Zięcina P (2015). Superselective renal artery embolization in the treatment of iatrogenic bleeding into the urinary tract. Med Sci Monit.

[4] El-Nahas AR, Shokeir AA, Mohsen T (2008). Functional and morphological effects of postpercutaneous nephrolithotomy superselective renal angiographic embolization. Urology.

[5] Thorlund MG, Wennevik GE, Andersen M, Andersen PE, Lund L (2015). High success rate after arterial renal embolisation. Dan Med J.

[6] Withington J, Neves JB, Barod R (2017). Surgical and minimally invasive therapies for the management of the small renal masses. Curr Urol Rep.

[7] Antonopoulos IM, Yamaçake KGR, Tiseo BC, Carnevale FC, Zieck E, Nahas WC (2016). Renal pseudoaneurysm after core-needle biopsy of renal allograft successfully managed with superselective embolization. Int Braz J Urol.

[8] Ahn T, Roberts MJ, Navaratnam A, Chung E, Wood S (2017). Changing etiology and management patterns for spontaneous renal hemorrhage: a systematic review of contemporary series. Int Urol Nephrol.

[9] Sam K, Gahide G, Soulez G (2011). Percutaneous embolization of iatrogenic arterial kidney injuries: safety, efficacy, and impact on blood pressure and renal function. J Vasc Interv Radiol.

[10] Guo H, Wang C, Yang M (2017). Management of iatrogenic renal arteriovenous fistula and renal arterial pseudoaneurysm by transarterial embolization: a single center analysis and outcomes. Medicine.

